# Efficacy of Neurocognitive Rehabilitation After Coronary Artery Bypass Graft Surgery in Improving Quality of Life: An Interventional Trial

**DOI:** 10.3389/fpsyg.2019.01759

**Published:** 2019-08-08

**Authors:** Simin Sadat Ajtahed, Tara Rezapour, Soraya Etemadi, Hadi Moradi, Mojtaba Habibi Asgarabad, Hamed Ekhtiari

**Affiliations:** ^1^College of Farabi, University of Tehran, Tehran, Iran; ^2^Department of Psychology, University of Tehran, Tehran, Iran; ^3^School of Electrical and Computer Engineering, University of Tehran, Tehran, Iran; ^4^Department of Health Psychology, School of Behavioral Sciences and Mental Health, Iran University of Medical Sciences, Tehran, Iran; ^5^Iranian National Center for Addiction Studies, Tehran University of Medical Sciences, Tehran, Iran; ^6^Translational Neuroscience Program, Institute for Cognitive Science Studies, Tehran, Iran; ^7^Research Center for Molecular and Cellular Imaging, Tehran University of Medical Sciences, Tehran, Iran

**Keywords:** computerized cognitive rehabilitation, coronary artery bypass graft surgery, cognitive functions, quality of life, improvement

## Abstract

**Introduction:**

Cognitive deficits are frequent after coronary artery bypass graft (CABG) surgery and consequently could lead to a decrease in quality of life. This is the first study that has been conducted with the aim of examining the efficacy of a computerized cognitive rehabilitation therapy (CCRT) in improving quality of life in patients after CABG surgery.

**Methods:**

In this study, an interventional trial with pre-, post-, and follow-up assessments in active (CCRT), active control and control groups was conducted. Seventy-five patients after CABG surgery were selected and assigned to the groups (*n* = 25 for each group). CCRT consists of four modules of attention, working memory, response inhibition and processing speed training with graded schedule in 20-min sessions three times per week within 8 weeks. Cognitive functions (attention and working memory) were assessed by the tests of continuous performance, Flanker, useful field of view and digit span at three time points: pre- and post-intervention (T0 and T1) and 6-month follow-up (T2). Quality of life was assessed by the SF-36 questionnaire at the same time points. The CCRT group received the cognitive rehabilitation for 2 months, active control group received a sham version of CCRT in an equal time duration and control group did not receive any cognitive intervention.

**Results:**

Repeated measures analysis of variance (ANOVA) revealed a time by group interaction on cognitive functions, with CCRT producing a significant improvement at T1 (*p* < 0.01) and these improvements were maintained at T2. Moreover, in CCRT and active control groups, quality of life (QoL) improved at T1 and these improvements remained stable throughout follow-up (T2). However, improvement of QoL in CCRT group was greater than improvement of QoL in the other two groups at T1. Pearson’s correlation analysis shows a positive correlation between QoL improvement and sustained attention and working memory enhancement (*p* < 0.05).

**Conclusion:**

Cognitive rehabilitation can lead to a significant improvement in the cognitive functions that have been trained in patients receiving CABG. Interestingly enough, cognitive rehabilitation can also improve quality of life in patients after CABG surgery and this improvement is maintained for at least 6 months.

## Introduction

Coronary artery disease (CAD) is the main cause of cardiovascular death worldwide (Okrainec et al., 2004) including United States (Benjamin et al., 2018), Europe (Townsend et al., 2016), and Iran (Hatmi et al., 2007). CAD is characterized by atherosclerosis in the pericardial coronary arteries. Atherosclerotic plaques can cause narrowing or blockage of coronary arteries and reducing blood flow and oxygen to the heart muscle (Cesar et al., 2014). Coronary artery bypass graft (CABG) surgery is one of the major treatment strategies for severe CAD (Yang et al., 2015); it is a surgical procedure to relieve angina (Samuel and Gopal, 2004). The aims of CABG surgery are to reduce previous symptoms of patients with coronary disease such as discomfort from chest pain (Yang et al., 2015), reduce the risk of heart attack, increase patient’s chance of living longer (Harris et al., 2013) and improve quality of life (QoL) (Järvinen et al., 2003; Panagopoulou et al., 2006; Kaur et al., 2013). World Health Organization (WHO) defines QoL as “the individual’s perception of his/her position in life, within the cultural context and the values in which he/she lives, as well as relation to his/her objectives, expectations, standards and concerns.” Thus, according to WHO, QoL includes of mental, physical and social well-being (The Whoqol, 1998). Therefore, the importance of the health-related QoL is significant in patients after the CABG surgery (Lee et al., 2015).

Beside all the benefits from CABG surgery, there are some evidences indicating that QoL is lower in patients after CABG surgery than healthy population (Marwick et al., 1999; Bradshaw et al., 2006; Rantanen et al., 2008). CABG surgery has different negative side effects. It is often associated with systemic inflammatory response and dysfunction of several organs, including brain; diffuse cerebral injury after CABG surgery may result in cognitive deficits (Kok et al., 2014). Also, some studies have shown that cerebral hypoperfusion occurs after cardiac surgery (Hogue et al., 2008), and it seems that there is relation between cerebral hypoperfusion and attention functions (Chokron et al., 2013).

Many previous studies have shown that cognitive deficits following CABG surgery include memory, attention, (psycho) motor speed, and visuospatial ability (Shaw et al., 1987; Hammeke and Hastings, 1988; Newman et al., 2001; Bruggemans, 2013; Goto and Maekawa, 2014). Selective and sustained attention and working memory decline are the most common cognitive deficits after CABG surgery and have been reported within 10 days after surgery (Toner, 1998; Westaby et al., 2001; Knipp et al., 2004). Cognitive deficits have been recognized as a significant complication after CABG surgery (Newman et al., 2001) and a risk factor for reduction in clinical health (Keizer et al., 2005). Cognitive deficits after CABG surgery have been associated with delayed postoperative recovery (McKhann et al., 1997), loss of independence, delayed return to work and impaired QoL (Bruce et al., 2013). The prevalence of short-term cognitive deficits (<2 weeks after surgery) has been estimated in 30–80% and long-term cognitive deficits (more than a month after surgery) in 10–60% of the patients (Rasmussen et al., 2001). Cognitive deficits can lead to significant damage to QoL in CABG patients after surgery (Kiessling and Henriksson, 2005; Phillips-Bute et al., 2006) by increasing risk for unemployment (Kiessling and Henriksson, 2005), hindering abilities to maintain social roles at a desirable level and poor function in daily life activities (Pan et al., 2015).

The association between cognitive deficits and QoL has been studied among different groups of patients including Alzheimer’s disease (AD), Parkinson’s disease, traumatic brain injury (TBI), and multiple sclerosis (MS) (Anderson et al., 2011; Leroi et al., 2012; Bosboom and Almeida, 2016; Camara et al., 2017). Previous studies have stated that cognitive deficits influence QoL by affecting the cognitive functions (such as memory) and reducing the learning skills and social and occupational performances (Barker-Collo et al., 2009; Ojeda et al., 2012).

However, despite all efforts made in the field of routine cardiac rehabilitation (RCR) (including medical evaluation, exercise training, cardiac risk factor, education and counseling) to improve the QoL (Hevey et al., 2003; Taylor et al., 2004; Aldena et al., 2006), some studies have stated that the proportion of patients who suffer from neurological complications has not changed and their QoL has not improved after RCR (Phillips-Bute et al., 2006; Cropsey et al., 2015). So we need a treatment that specifically focuses on cognitive deficits. Cognitive rehabilitation therapy (CRT) has a long and successful history in the field of TBI, schizophrenia and AD (Salazar et al., 2000; Velligan et al., 2006; Choi and Twamley, 2013). CRT is the science of compensating cognitive processing; it can result to the molecular and cellular recovery rehabilitation by integrating the behavioral and cognitive changes (Zarghi, 2014). CRT is used to improve cognitive functions including attention, memory and decision-making (Rezapour et al., 2016). CRT consists of two components: restorative exercises and compensatory strategies (Samuel, 2008). The aim of restorative exercise is to restore the impaired cognitive functions by using computerized or paper-pencil exercises. While, the aim of compensatory strategy is to train patients how to use external tools to compensate their cognitive impairments (Barman et al., 2016). Computerized training programs have several advantages over paper-based training tools. For example, they can increase patient’s motivation for treatment because of direct feedback and reducing treatment duration (Yoo et al., 2015; Politis and Norman, 2016).

There is a study that has investigated the efficacy of CRT as a treatment to improve cognitive deficits after CABG surgery. Neuropsychological battery was used to assess cognitive changes following cardiac surgery. Training sessions included attention and working memory training tasks to improve divided attention and memory. Findings showed significant impact of trainings on attention and memory. Also, improvement of some cognitive functions that were not trained during intervention was reported (de Tournay-Jette et al., 2012). But, some questions have remained unanswered. For example, it is still unclear if improving cognitive functions would lead to improving QoL in CABG patients after surgery or not.

Therefore, the present study has been conducted to assess the efficacy of using computerized cognitive rehabilitation therapy (CCRT) as a supplementary treatment to RCR in patients after CABG surgery in terms of both cognitive functioning and QoL. Although there are some studies that have suggested another strategies to improve QoL in CABG patients such as optimizing patients’ expectations (Rief et al., 2017), the hypothesis of this study was that patients who receive RCR plus CCRT would show greater improvement in cognitive functions and QoL than those who receive RCR plus sham version of CCRT and who receive RCR without any cognitive intervention.

## Materials and Methods

### Participants

In this experimental study, a three-armed interventional trial with pre-, post-, and follow-up assessments in CCRT, active control and control groups was conducted. This study was a single blind trial in which 75 patients (male = 50 and female = 25) enrolled after CABG surgery between October 2017 and September 2018 in Tehran Heart Center. Allocation was carried out by the patient enrolment date (the first month = CCRT group, the second month = active control group, and the third month = control group). Before starting RCR, general assessment of patients (including demographic and clinical data) was recorded and patients completed the QoL questionnaire (SF-36), then baseline assessment of cognitive functions was done using Continuous performance, Flanker, Useful Field of View, and Digit Span tests. During the intervention period, control group only received RCR, active control group received RCR plus sham version of computerized cognitive training program and CCRT group received RCR plus computerized cognitive training program.

Patients were included in the study if they were 40–80 years of age, scheduled for a first CABG surgery without concomitant surgery, capable of perception of the research purpose, able to give informed consent before any study, able to speak in Persian with study personnel, able to read and write and work with computer and participate in concurrent standard routine rehabilitation during the trial. Patients were excluded if they used any medications that could have any effect on the cognitive functions (all antihypertensives and diuretics, antidepressants and anticonvulsants), had previous or current drug or alcohol abuse and had a mental disorder (major depression, schizophrenia or bipolar disorder) due to their negative effects on cognitive functions (Wetherell et al., 2002; Sheline et al., 2006; Sandi, 2013) and QoL (Gudmundsdottir et al., 2004). It should be mentioned that a structured clinical interview was used to screen for mental disorders by a certified psychiatrist. This study was approved by Ethics Committee of the Iranian Ministry of Health (reference number: IR.ut.Rec.1395029) and the Iranian Registry of Clinical Trials (IRCT ID: IRCT2017022132704N1) (https://irct.ir/trial/25408).

### Intervention

Patients in CCRT group received CCRT plus RCR. CCRT was conducted individually in 24, 20-min sessions within 8 weeks. The cognitive training program applied in this study was Maghzineh®; Maghzineh^®^ is a computerized training for cognitive rehabilitation that activates different cognitive functions including attention, working memory, and inhibition.

**FIGURE 1:**
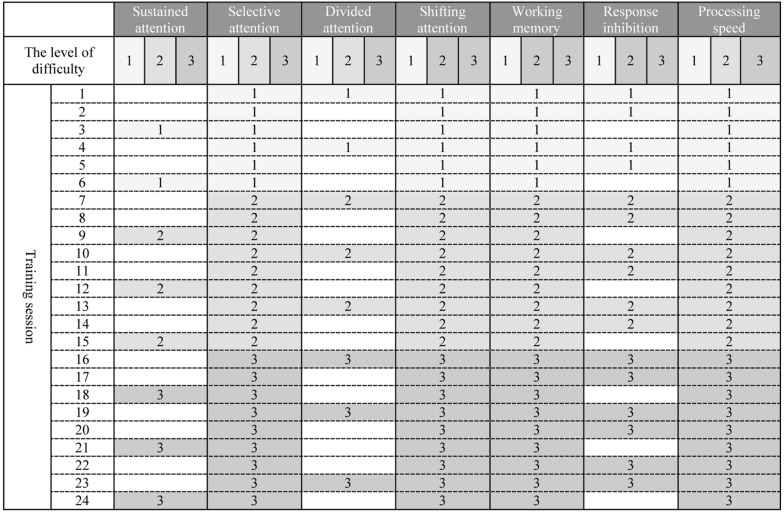
(Architecture) Graded schedule of computerized cognitive rehabilitation exercises for 24 sessions with Maghzineh®. Numbers indicate the level of difficulty of each session exercises (1 = the easiest level to 3 = the hardest level).

Maghzineh^®^ developed in 24 sessions. Generally the level of difficulty for each task increases gradually (see Figure 1) but according to the patient’s performance the pattern shown in Figure 1 might be modified.

Patients in the active control group received a sham version of CCRT plus RCR. The sham version involved the same training program as Maghzineh^®^ without adaptivity and the difficulty level remained constant across the entire intervention. Active control intervention was chosen to reduce the probable confounding effects of social interaction of the CCRT.

Patients in the control group received RCR without any cognitive intervention for 2 months.

### Assessments

Assessments were performed at three time points: pre-, post-intervention, and 6-month follow-up.

#### Neuropsychological Assessments

Cognitive functions (including sustained, selective and divided attention and working memory) were evaluated by the following assessments:

• Continues Performance Test (CPT): This is a test to measure sustained attention which means the ability to keep attention focused over time (Mirsky et al., 1991). The version used in this study has 50 digits as stimulus in which 10 stimuli (20%) are targets. Display time of each stimulus was 150 ms and interval between stimuli was 200 and 500 ms, respectively. Patients were presented a simple set of visual stimuli (random digits from 1 to 9) in specific period of time and were asked to respond to target stimulus (9 number) as quickly as possible by pressing the space bar once. This test measures the time of the response as well as its accuracy.
• Flanker Test (FT): This test was designed in the early 1970s by Eriksen and Eriksen. The FT assesses the selective attention and response inhabitation (Eriksen and Eriksen, 1974). Selective attention allows to successfully focus on goal relevant information while inhibiting irrelevant information. As such selective attention is part of inhibitory control (Reuter et al., 2019). In this study, patients were presented 80 sets of arrows as a stimulus (central arrow as target stimulus surrounded by other distractor arrows). In the first step, patients were asked to focus on the center of computer screen and press direction arrow on the keyboard of computer that its direction is the same as central arrow on the screen. In second step, patients were asked to press opposite direction arrow key with central arrow on the screen.
• Useful Field of View Test (UFoVT): UFoV is a test to assess divided attention (Gray et al., 2014). Patients had to detect, identify and localize briefly presented targets. The patients had to and perform the central and peripheral tasks concurrently. Patients were asked to identify the type of vehicle (truck or car) appeared in the center of the screen and the one displayed in the periphery of the screen at one of eight possible radial locations (0°, 45°, 90°, 135°, 180°, 225°, 270°, and 315°) around the center (It should be noted that the type of vehicle in the center and periphery were not necessarily the same).
• Digit Span Test (DST): The DST measures working memory and has two subsections: forward digit span (FDS) and backward digit span (BDS) (Goghari and Lawlor-Savage, 2017). In the FDS subsection, patients were presented with a series of digits on the computer screen; each trial includes a sequence of digits (varied from 2–8) flashing at once. Patients were asked to repeat the sequence in the same order, immediately after presentation. A correct response increases the length of the subsequent list by one digit, while two sequential incorrect responses end up the test. In the BDS, the patient was asked to choose the sequence in reverse order.

#### Quality of Life Assessment

QoL Short Form (SF-36) questionnaire: This test was designed in the 1992s by Ware and Sherbourne. The SF-36 questionnaire asks 36 questions related to an individual’s functional health and well-being (Ware and Sherbourne, 1992). The SF-36 assesses eight aspects of QoL, including physical functioning (PF), role physical (RP), bodily pain (BP), general health (GH), vitality (VT), social functioning (SF), role emotional (RE), and mental health (MH) (Qu et al., 2009). Scores were calculated for each of these different aspects of health in the range 0–100, low score indicating poor health status (Talamo et al., 1997). The reliability of the Persian version of this questionnaire has been confirmed by using Cronbach’s alpha from 0.77 to 0.90 (Montazeri et al., 2005).

#### Clinical Assessments

After admission, the cardiac rehabilitation team including doctors, nurses, nutritionists and mental health specialists recorded demographic and clinical data (Diabetic Mellitus, Hypertension, Family History, and Hyperlipidemia). Since many evidences suggested that patients experience depression, anxiety and stress after CABG surgery (Tully and Baker, 2012; Chaudhury et al., 2016), the DASS scale was used for comparison of groups in baseline. In a study conducted in Iran with 378 non-clinical adults, Cronbach’s alpha for Depression, Anxiety and Stress scales were 0.85, 0.85, and 0.87, respectively (Asghari et al., 2008).

### Statistical Analysis

Based on a *p*-value of 0.05, an expected drop-out from assessments of 50%, the study had a power of 80%, and an effect size of 0.25 (medium), a group of 75 subjects defined as a sample size. Descriptive statistics were used to describe the baseline characteristics of participants. Kruskal–Wallis *H* test was conducted to assess baseline differences of non-parametric characteristics of patients in three groups, analysis of variance (ANOVA) was used for parametric characteristics. Also, analysis of correlation was used to assess relationship between QoL and cognitive functions in baseline among all participants. The assumption of normal distribution of all parameters was examined with Kolmogorov–Smirnov, as a result the significance level of all parameters was ≥0.05, indicating a normal distribution. Repeated ANOVAs were performed to evaluate the effect of group (CCRT vs. control vs. active control), time (T0 vs. T1 vs. T2) and the time by group interaction, including only patients who accomplished the whole clinical trial according to the protocol. To estimate the effect size of intervention, partial eta squared values ($\eta_{p}^{2}$) were used and Pearson correlation analysis was conducted to illuminate the relationship between QoL improvement and cognitive functions enhancement among all participants. The QoL improvement and cognitive functions enhancement were calculated by subtracting the scores of QoL and cognitive functions in pre from Follow-up assessment. Where significant effects were found, pairwise comparison were carried out using the Bonferroni correction. Results of the *post hoc* comparison were presented as mean difference (MD) with 95% confidence interval (CI) and standard error (SE). Finally, an expectation maximization (EM) algorithm was performed to take the missing data into account. All statistical analysis was performed using SPSS v.23.0. Data for this study is pre-registered and accessible on Open Science Framework (OSF), titled “Efficacy of Neurocognitive Rehabilitation After Coronary Artery Bypass Graft Surgery in Improving Quality of Life.” https://osf.io/m249v.

## Results

### Baseline Characteristics

**FIGURE 2:**
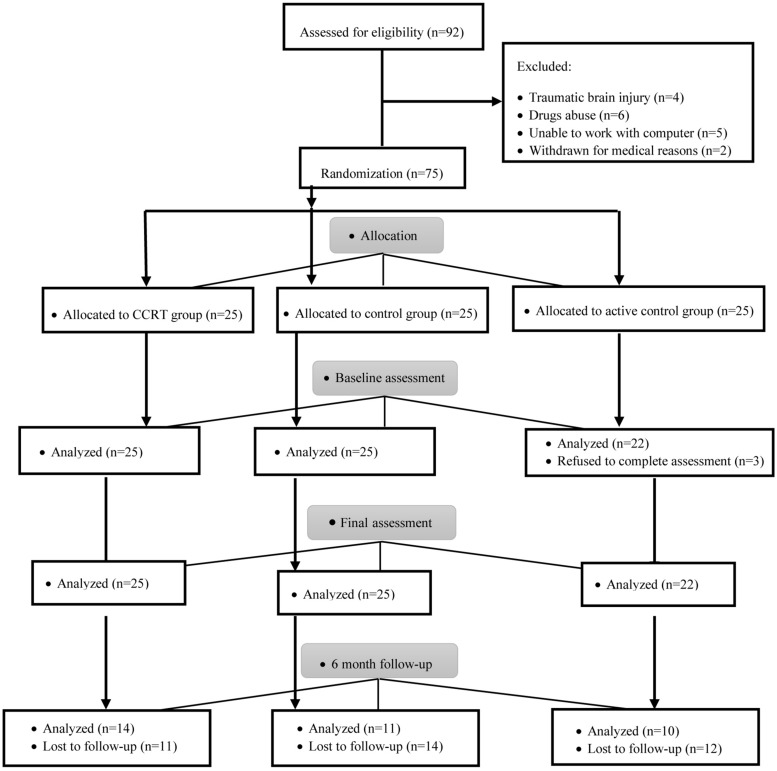
CONSORT flow diagram of patients through the study.

Patient flow is presented in Figure 2. A total of 75 participants after CABG surgery were registered and assigned 1:1:1 to CCRT, active control and control groups.

Baseline analysis showed no significant difference in terms of demographic variables (*p* > 0.05), clinical variables (*p* > 0.05), cognitive functions (*p* > 0.05), and QoL (*p* > 0.05) measures between the three groups (Table 1) and there was no significant correlation between QoL and cognitive functions among all participants (sustained attention: Pearson’s *r* = 0.00, *p* = 0.958; selective attention: Pearson’s *r* = 0.11, *p* = 0.331; divided attention: Pearson’s *r* = 0.03, *p* = 0.781; working memory: Pearson’s *r* = 0.08, *p* = 0.488). In addition, a comparison of completers and drop-outs in all three groups found no baseline difference in the demographical and clinical characteristics of those assigned to intervention.

**TABLE 1:** Demographics, clinical variables and baseline scores of cognitive measures and QoL questionnaire in CCRT, control and active control groups.

**Components**			**CCRT group (*n* = 25)**	**Control group (*n* = 25)**	**Active Control group (*n* = 22)**	***P*-value**
Age mean (SD)			56.96 (15.20)	56.48 (12.73)	57.95 (9.76)	*p* = 0.94
Sex *n* (%)		Male	15 (60)	20 (80)	14 (63.6)	*p* = 0.28
		Female	10 (40)	5 (20)	8 (36.4)	
Education *n* (%)		Primary school	5 (20)	8 (32)	7 (31.8)	*p* = 0.56
		High school	14 (56)	10 (40)	12 (54.5)	
		University	6 (24)	7 (28)	3 (13.6)	
Marital status *n* (%)		Single	3 (12)	3 (12)	3 (13.6)	*p* = 0.98
		Married	22 (88)	22 (88)	19 (86.4)	
DM *n* (%)		Yes	8 (32)	14 (56)	6 (27.3)	*p* = 0.09
		No	17 (68)	11 (44)	16 (72.7)	
HTN *n* (%)		Yes	8 (32)	9 (36)	6 (27.3)	*p* = 0.82
		No	17 (68)	16 (64)	16 (72.7)	
FH *n* (%)		Yes	2 (8)	7 (28)	6 (27.3)	*p* = 0.15
		No	23 (92)	18 (72)	16 (72.7)	
HLP *n* (%)		Yes	4 (16)	8 (32)	3 (13.6)	*p* = 0.23
		No	21 (84)	17 (68)	19 (86.4)	
Obesity *n* (%)		Yes	3 (12)	1 (4)	0	*p* = 0.19
		No	22 (88)	24 (96)	22 (100)	
Personality *n* (%)		Type A	17 (68)	22 (88)	14 (63.6)	*p* = 0.12
		Type B	8 (32)	3 (12)	8 (36.4)	
Smoker *n* (%)		Yes	5 (20)	7 (28)	4 (18.2)	*p* = 0.69
		No	20 (80)	18 (72)	18 (81.1)	
DASS	Depression *n* (%)	Normal	15 (60)	13 (52)	10 (45)	*p* = 0.38
		Mild	1 (40)	2 (8)	2 (9.1)	
		Moderate	6 (24)	8 (32)	3 (13.6)	
		Severe	3 (12)	0	3 (13.6)	
		Extremely severe	0	2 (8)	4 (18.2)	
	Anxiety *n* (%)	Normal	11 (44)	9 (36)	7 (31.8)	*p* = 0.51
		Mild	3 (12)	5 (20)	4 (18.2)	
		Moderate	7 (28)	2 (8)	3 (13.6)	
		Severe	1 (4)	5 (20)	2 (9.1)	
		Extremely severe	3 (12)	4 (16)	6 (27.3)	
	Stress *n* (%)	Normal	11 (44)	9 (36)	11 (50)	*p* = 0.55
		Mild	3 (12)	4 (16)	1 (4.5)	
		Moderate	7 (28)	9 (36)	1 (4.5)	
		Severe	1 (4)	2 (8)	6 (27.3)	
		Extremely severe	3 (12)	1 (4)	3 (13.6)	
Cognitive functions mean (SD)		Continues performance	58.74 (9.39)	59.58 (10.88)	65.25 (10.66)	*p* = 0.07
		Flanker	84.90 (13.58)	81.74 (16.44)	82.72 (13.24)	*p* = 0.73
		Useful field of view	86.86 (11.77)	89.21 (11.81)	81.20 (16.04)	*p* = 0.11
		Forward digit span	73.96 (8.12)	73.76 (9.60)	70.18 (10.90)	*p* = 0.32
		Backward digit span	66.52 (13.46)	67.72 (7.66)	64.95 (6.64)	*p* = 0.63
QoL mean (SD)			53.48 (12.21)	49.70 (18.38)	44.22 (18.29)	*p* = 0.26

*No significant differences between groups were observed. DM = Diabetic Mellitus; HTN = Hypertension; FH = Family History; HLP = Hyperlipidemia; DASS = Depression Anxiety Stress Scales.*

### Primary Outcomes in Final Assessment

The repeated ANOVA was carried out; in the Table 2 the main effect of time, intervention and time by group interaction as well as mean scores corresponding to time points is presented. Furthermore in the Table 3 pairwise comparison results are reported according to Bonferroni correction.

**TABLE 2:** Summary of pre- and post-intervention and 6-month follow-up measures and significant interaction on repeated ANOVA.

**Components**	**CCRT group (*n* = 14)**			**Control group (*n* = 11)**			**Active Control group (*n* = 10)**			**Intervention F (*p*) [$\eta_{p}^{2}$]**	**Time F (*p*) [$\eta_{p}^{2}$]**	**Intervention × time *F* (*p*) [$\eta_{p}^{2}$]**
			
	**T0**	**T1**	**T2**	**T0**	**T1**	**T2**	**T0**	**T1**	**T2**						
Sustained attention	57.36	72.32	71.20	58.60	69.14	61.21	64.66	62.26	62.55	0.92	10.85	6.77
	(10.37)	(9.08)	(10.01)	(8.78)	(6.54)	(14.03)	(11.02)	(9.12)	(9.75)	(*p* = 0.406)	(*p* < 0.001)	(*p* < 0.001)
										[0.05]	[0.25]	[0.29]
Selective attention	83.21	99.19	98.28	81.45	85.83	84.63	85.25	83.00	82.50	2.96	7.25	6.72
	(16.81)	(0.79)	(1.54)	(20.21)	(16.56)	(15.93)	(9.57)	(11.16)	(11.67)	(*p* = 0.066)	(*p* = 0.008)	(*p* = 0.002)
										[0.15]	[0.18]	[0.29]
Divided attention	84.99	91.66	98.07	88.98	86.36	87.63	80.49	81.99	79.40	2.30	3.85	8.19
	(13.44)	(10.84)	(2.16)	(13.03)	(13.75)	(11.64)	(18.77)	(14.67)	(16.47)	(*p* = 0.117)	(*p* = 0.043)	(*p* < 0.001)
										[0.12]	[0.10]	[0.33]
Working memory	69.53	86.35	84.96	69.68	67.95	64.13	65.85	64.30	66.55	36.30	6.47	16.69
	(7.93)	(4.21)	(6.10)	(5.99)	(8.04)	(6.66)	(5.41)	(5.02)	(8.04)	(*p* < 0.001)	(*p* = 0.003)	(*p* < 0.001)
										[0.69]	[0.16]	[0.51]
QoL	48.03	73.44	74.10	45.20	53.52	55.01	41.50	50.13	50.75	4.74	45.88	7.39
	(17.69)	(14.48)	(12.90)	(18.06)	(18.83)	(13.98)	(20.98)	(14.90)	(13.20)	(*p* = 0.016)	(*p* < 0.001)	(*p* = 0.001)
										[0.22]	[0.58]	[0.31]

*Values represent mean scores (SD and $\eta_{p}^{2}$ are between parenthesis and brackets respectively). Significant time by group interaction was observed. T0 = pre-intervention (week 0); T1 = post-intervention (week 8); T2 = 6-month follow-up (week 32).*

**TABLE 3:** Summary of *post hoc* analysis (Bonferroni correction).

**Components**	**Groups**		**MD**	**SE**	***p*-value**	**95% CI**	
					
						**Lower**	**Upper**
Sustained attention	CCRT	Control	3.97	3.33	0.72	–4.45	12.40
		Active control	3.80	3.43	0.82	–4.86	12.46
	Control	Active control	–0.17	3.61	1.00	–9.31	8.97
Selective attention	CCRT	Control	9.59	4.69	0.14	–2.27	21.45
		Active control	9.98	4.82	0.14	–2.20	22.17
	Control	Active control	0.39	5.09	1.00	–12.47	13.25
Divided attention	CCRT	Control	3.91	497	1.00	–8.65	16.49
		Active control	10.94	5.11	0.12	–1.97	23.87
	Control	Active control	7.02	5.39	0.60	–6.60	20.66
Working memory	CCRT	Control	13.02	1.90	0.00	8.21	17.84
		Active control	14.71	1.95	0.00	9.77	19.66
	Control	Active control	1.69	2.06	1.00	–3.52	6.90
QoL	CCRT	Control	13.94	6.07	0.08	–1.40	29.29
		Active control	17.72	6.24	0.02	1.95	33.50
	Control	Active control	3.78	6.58	1.00	–12.86	20.43

*Based on estimated marginal means. Adjustment for multiple comparisons: Bonferroni.*

#### Sustained Attention

**FIGURE 3:**
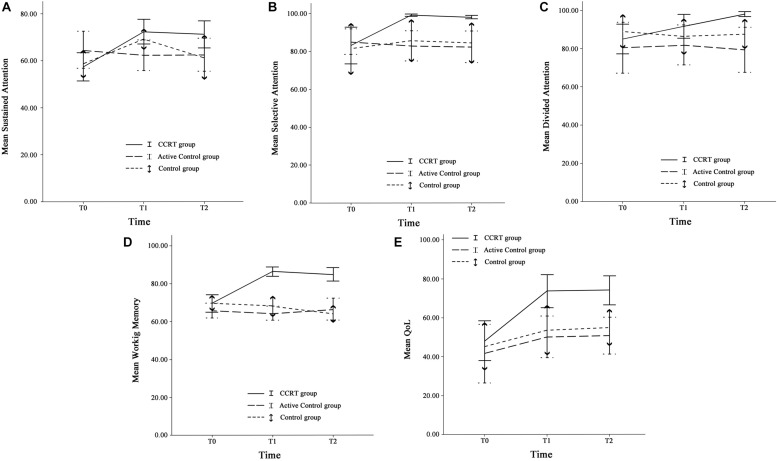
Cognitive functions and QoL. **(A)** Sustained attention, **(B)** Selective attention, **(C)** Divided attention, **(D)** Working memory, and **(E)** QoL at three time points. Error bars represent 95% confidence interval (CI). T0 = pre-intervention (week 0); T1 = post-intervention (week 8); T2 = 6-month follow-up (week 32).

Findings of present study implied that there was time effect on sustained attention as well as a large-sized intervention by time interaction (*p* < 0.001, $\eta_{p}^{2}$ = 0.29). Compared with baseline, sustained attention in CCRT and control groups improved at T1 (CCRT group: MD = 14.95, 95%CI = [−25.18, −4.71], SE = 3.72, *p* = 0.004; control group: MD = 10.54, 95%CI = [−17.78, −3.31], SE = 2.52, *p* = 0.006). In both groups the improvement remained stable at the follow-up (CCRT group: MD = −1.11, 95%CI = [−4.42, 6.66], SE = 2.01, *p* = 1.000; Control group: MD = −7.93, 95%CI = [−1.18, 17.04], SE = 3.17, *p* = 0.095) (Figure 3). There was no effect of group on sustained attention.

#### Selective Attention

There was large-sized effect of time on selective attention score and a large-sized intervention by time interaction (*p* = 0.002, $\eta_{p}^{2}$ = 0.29). Selective attention in the CCRT group, but not in the other two groups, improved at T1 relative to baseline (MD = 15.98, 95%CI = [4.03, 27.92], SE = 4.35, *p* = 0.008) and this improvement remained stable at T2 (MD = −0.91, 95%CI = [−1.87, 0.05], SE = 0.35, *p* = 0.068) (Figure 3). There was no effect of group on selective attention.

#### Divided Attention

There was time effect on divided attention score also large-sized effect group by time interaction (*p* = 0.00, $\eta_{p}^{2}$ = 0.33), reflecting the lack of change in divided attention in control and active control groups (control group: T0 vs. T1: MD = −2.62, 95%CI = [−7.18, 1.93], SE = 1.58, *p* = 0.390; T1 vs. T2: MD = 1.27, 95%CI = [−2.80, 5.35], SE = 1.42, *p* = 1.000; active control group: T0 vs. T1: MD = 1.49, 95%CI = [−2.77, 5.77], SE = 1.45, *p* = 0.993; T1 vs. T2: MD = −2.59, 95%CI = [−6.65, 1.46], SE = 1.38, *p* = 0.281). In contrast, the CCRT improved divided attention score at T1 (MD = 6.66, 95%CI = [3.34, 9.99], SE = 1.21, *p* < 0.001) and this improvement remained stable at T2 (MD = 6.40, 95%CI = [−1.36, 14.18], SE = 2.83, *p* = 0.124) (Figure 3). There was no effect of group on divided attention.

#### Working Memory

There were group and time effects on working memory score. There was large-sized effect group by time interaction (*p* < 0.001, $\eta_{p}^{2}$ = 0.51). Pairwise comparisons confirmed that the CCRT led to improvement of working memory score at T1 relative to baseline (MD = 16.82, 95%CI = [10.71, 22.92], SE = 2.22, *p* < 0.001) that was maintained at T2 (MD = −1.39, 95%CI = [−6.13, 3.34], SE = 1.72, *p* = 1.000) (Figure 3). Pairwise comparison revealed that the working memory improvement in CCRT group was significantly greater than the working memory improvement in other two groups.

### QoL Outcomes in Final Assessment

Table 2 presents QoL mean scores corresponding to pre-, post-intervention and 6-month follow-up measurements. There were time and group effects on QoL and Large-sized effect group by time interaction (*p* = 0.001, $\eta_{p}^{2}$ = 0.31). Pairwise comparison confirmed that in CCRT and active control groups, but not in control group, QoL increased at T1 relative to T0 (CCRT group: MD = 25.41, 95%CI = [14.93, 35.89], SE = 3.81, *p* < 0.001; active control group: MD = 8.62, 95%CI = [0.51, 16.74], SE = 2.76, *p* = 0.037). In both groups the improvement remained stable at T2 (CCRT group: MD = 0.65, 95%CI = [−1.43, 2.74], SE = 0.76, *p* = 1.000; active control group: MD = 0.61, 95%CI = [−2.04, 3.27], SE = 0.90, *p* = 1.000). However, pairwise comparison revealed that the QoL improvement in CCRT group was significantly greater than the QoL improvement in active control group (MD = 17.72, 95%CI = [15.18, 36.55], SE = 6.24, *p* = 0.023) (Figure 3).

### Correlation Between QoL Improvement and Cognitive Functions Enhancement

**FIGURE 4:**
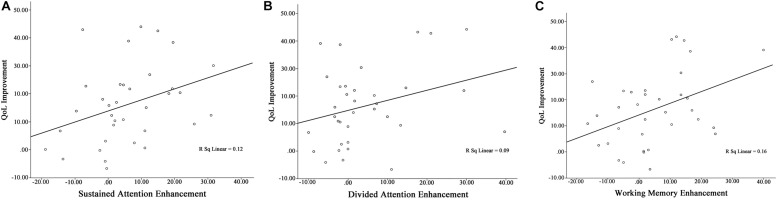
Pre/follow-up correlation between **(A)** QoL improvement and sustained attention enhancement, **(B)** QoL improvement and divided attention enhancement, and **(C)** QoL improvement and working memory enhancement.

All patients were included in the analysis of correlation Table 4 shows relationship between QoL improvement and cognitive functions enhancement. Findings of current study shows significant correlation between QoL improvement and cognitive functions (except selective and divided attention) enhancement (sustained attention: Pearson’s *r* = 0.34, *p* = 0.041; selective attention: Pearson’s *r* = 0.32, *p* = 0.057; divided attention: Pearson’s *r* = 0.30, *p* = 0.078; working memory: Pearson’s *r* = 0.40, *p* = 0.016) (Figure 4).

**TABLE 4:** Summary of pre/follow-up correlation between QoL improvement and cognitive functions enhancement.

**QoL improvement**	**Cognitive functions**							
	
	**Sustained attention (*n* = 35)**		**Selective attention (*n* = 35)**		**Divided attention (*n* = 35)**		**Working memory (*n* = 35)**	
				
	**Pearson’s *r***	***p*-value**	**Pearson’s *r***	***p*-value**	**Pearson’s *r***	***p*-value**	**Pearson’s *r***	***p*-value**
	0.34^*^	0.041	0.32	0.057	0.30	0.078	0.40^*^	0.016

^*^Correlation is significant at the 0.05 level.

### EM Algorithm

The EM algorithm is a general method for obtaining maximum likelihood estimates of parameters from incomplete data. The EM algorithm is based on the intuitive idea of estimating the missing value and iteratively re-estimating the parameters using the estimated missing values (Imtiaz and Shah, 2008). Thirty-seven patients (51%) from three groups refused to complete the assessments at T2. Statistical analysis showed no significant difference in terms of demographic variables (*p* > 0.05), clinical variables (*p* > 0.05), cognitive functions (*p* > 0.05), and QoL (*p* > 0.05) measures between drop-out and remained patients. This indicates a random pattern of missing values.

In the EM algorithm there were main effects of time and group as well as the time by group interaction for all outcome scores (sustained attention: *p* < 0.001, $\eta_{p}^{2}$ = 0.23; selective attention: *p* < 0.001, $\eta_{p}^{2}$ = 0.27; divided attention: *p* < 0.001, $\eta_{p}^{2}$ = 0.28; working memory: *p* < 0.001, $\eta_{p}^{2}$ = 0.47; QoL: *p* < 0.001, $\eta_{p}^{2}$ = 0.27) (Table 5).

**TABLE 5:** Summary of repeated ANOVA on the entire patients (EM algorithm).

**Components**	**CCRT group (*n* = 25)**			**Control group (*n* = 25)**			**Active Control group (*n* = 22)**			**Intervention F (*p*) [$\eta_{p}^{2}$]**	**Time F (*p*) [$\eta_{p}^{2}$]**	**Intervention × time *F* (*p*) [$\eta_{p}^{2}$]**
						
	**T0**	**T1**	**T2**	**T0**	**T1**	**T2**	**T0**	**T1**	**T2**						
Sustained attention	58.74	71.20	69.94	59.58	63.43	58.63	65.25	64.34	63.07	3.24	11.86	10.52
	(9.39)	(7.32)	(8.34)	(10.88)	(10.43)	(12.44)	(10.66)	(9.27)	(9.70)	(*p* = 0.045)	(*p* < 0.001)	(*p* < 0.001)
										[0.08]	[0.14]	[0.23]
Selective attention	84.90	98.95	98.11	81.74	85.81	85.45	82.72	81.64	81.24	8.21	20.52	13.22
	(13.58)	(0.93)	(1.74)	(16.44)	(12.80)	(12.33)	(13.24)	(13.11)	(13.36)	(*p* = 0.001)	(*p* < 0.001)	(*p* < 0.001)
										[0.19]	[0.22]	[0.27]
Divided Attention	86.86	93.26	98.33	89.21	86.19	87.82	81.20	81.90	80.89	6.70	7.92	14.00
	(11.77)	(8.95)	(3.36)	(11.81)	(12.31)	(10.13)	(16.04)	(12.85)	(12.62)	(*p* = 0.002)	(*p* = 0.01)	(*p* < 0.001)
										[0.16]	[0.10]	[0.28]
Working memory	70.24	84.76	83.49	70.74	68.78	66.07	67.56	64.70	65.75	57.37	7.90	31.46
	(6.94)	(4.44)	(5.33)	(6.18)	(7.77)	(6.58)	(5.83)	(5.44)	(6.39)	(*p* < 0.001)	(*p* = 0.001)	(*p* < 0.001)
										[0.62]	[0.10]	[0.47]
QoL	53.48	77.71	77.18	49.70	60.56	60.79	44.22	51.84	52.26	10.98	106.53	12.91
	(21.12)	(13.41)	(11.65)	(18.38)	(16.44)	(12.84)	(18.29)	(14.62)	(13.01)	(*p* < 0.001)	(*p* < 0.001)	(*p* < 0.001)
										[0.24]	[0.60]	[0.27]

*Values represent mean scores (SD and $\eta_{p}^{2}$ are between parenthesis and brackets respectively). Significant time by group interaction was observed. T0 = pre-intervention (week 0); T1 = post-intervention (week 8); T2 = 6-month follow-up (week 32).*

## Discussion

The purpose of this study was to examine the efficacy of a CCRT in a group of patients after CABG surgery. We sought to find whether using cognitive rehabilitation could improve the QoL in this group of patients. According to what we have found, significant QoL improvement was detected in patients who received CCRT compared to the other two groups. In other words, our results support the beneficial effects of incorporation of cognitive rehabilitation in the RCR. This is one of the first studies to demonstrate the effectiveness of using cognitive rehabilitation after CABG surgery.

The positive association between cognitive rehabilitation and the improved QoL has been previously reported in patients with MS, Parkinson’s disease and brain tumors (Locke et al., 2008; Paris et al., 2011; Mitolo et al., 2015; Grasso et al., 2017). But less is known about the patients who receive CABG surgery, while a large body of evidence reports postoperative cognitive deficits in this group of patients (Shaw et al., 1987; Hammeke and Hastings, 1988; Newman et al., 2001; Bruggemans, 2013; Goto and Maekawa, 2014).

We used a computerized cognitive rehabilitation program for one group of patients which resulted in improved cognitive functions in terms of sustained, selective, divided attention and working memory. This result extends the previous work by de Tournay-Jette et al. (2012) by using a larger sample size. In our study, those patients who receive CCRT within 2 months, outperformed the other two groups on different tests of attention and working memory. This suggests that the applied program was an efficient cognitive training for our sample that could help them to engage and disengage their attention properly in the context of real life.

Moreover, a significant improvement in sustained attention was found for our control group but not for the active control group. This shows that the sham version used for the active control group, didn’t provide any targeted training.

Consistent with previous studies, improved cognitive functions is associated with better QoL (Fabre et al., 2002; Prasad et al., 2007; Woods et al., 2012). This is because cognitive impairments can reduce daily function and social interactions of individuals and make them more dependent on others for their everyday activities (Kiessling and Henriksson, 2005; Ojeda et al., 2012; Pan et al., 2015; Saraçlı et al., 2015). Therefore, it is no surprising that QoL was reported higher by CCRT group compared to the other two groups. While the active control group achieved the same positive results as the CCRT group, the CCRT group rated QoL significantly better than the active control group. With regard to the possibility of chance findings due to the high rate of drop-out at follow-up, it is very important for future investigations to carry out the similar study with larger sample size.

Taken together, these results suggest the benefits of adding cognitive rehabilitation as an adjunct therapeutic intervention for patients undergoing CABG surgery to ameliorate postoperative cognitive deficits and increase QoL. These results offer a new line of research for developing a new cognitive rehabilitation tool that specifically targets postoperative cognitive deficits that are commonly observed in this group.

Our study has several strengths. We have used a 3-arm interventional design that allowed us to compare three different conditions. There was also a 6-month follow-up to track the training benefits over time. But, there are also some limitations that should be noted. First, the CCRT used in this study was focused on attention and working memory, so further studies would benefit from targeting other cognitive functions including executive functions (Rezapour et al., 2017). Second, we didn’t consider the far transfer effects of training in real life situations (e.g., managing household, managing medications), thus we suggest to use relevant measures for better understanding the effects of cognitive rehabilitation. Another limitation of this study is related to the high rate of drop-out at 6-month follow-up that should be considered and controlled in future studies. Finally, our limitation to access patient’s medical record was a barrier to consider them in our analysis, so including these data in future investigations allows detailed comparison of the baseline characteristics.

In conclusion, the results obtained in this study with regard to improvement of cognitive functions as well as QoL in patients after CABG surgery, may follow valuable clinical implications for those who provide health care services for this group of patients.

## Ethics Statement

This study was carried out in accordance with the recommendations of Ethics Committee of the Iranian Ministry of Health (reference number: IR.ut.Rec.1395029) with written informed consent from all patients. All patients gave written informed consent in accordance with the Declaration of Helsinki. The protocol was approved by the Iranian Registry of Clinical Trials, IRCT ID: IRCT2017022132704N1 (http://www.irct.ir/trial/25408).

## Author Contributions

HE, SA, TR, HM, and SE were responsible for the conception and design of the study. SA and SE were responsible for data collection and for evaluations and treatments. MH was responsible for data analysis. TR and SA contributed to the interpretation of data. SA wrote the manuscript, which was critically revised by HE and TR. All authors have approved the final version of the manuscript.

## Conflict of Interest Statement

HE and TR designed the Maghzineh architecture and games. HM is the CEO of Pars Cognition, a small spin off company in the University of Tehran incubator. The remaining authors declare that the research was conducted in the absence of any commercial or financial relationships that could be construed as a potential conflict of interest.

## References

[B1] Aldena S. G. Whitmer W. R. Greenlaw R. Avins A. L. Thomas D. Salberg A. (2006). Effect of intense lifestyle modification and cardiac rehabilitation on psychosocial cardiovascular disease risk factors and quality of life. *Behav. Modif.* 30 507–525. 10.1177/0145445504267797 16723428

[B2] Anderson V. Brown S. Newitt H. Hoile H. (2011). Long-term outcome from childhood traumatic brain injury: intellectual ability, personality, and quality of life. *Neuropsychology* 25 176–184. 10.1037/a0021217 21219074

[B3] Asghari A. Saed F. Dibajnia P. (2008). Psychometric properties of the depression anxiety stress scales-21 (DASS-21) in a non-clinical Iranian sample. *Int. J. Psychol.* 2 82–102.

[B4] Barker-Collo S. L. Feigin V. L. Lawes C. M. Parag V. Senior H. Rodgers A. (2009). Reducing attention deficits after stroke using attention process training: a randomized controlled trial. *Stroke* 40 3293–3298. 10.1161/STROKEAHA.109.558239 19628801

[B5] Barman A. Chatterjee A. Bhide R. (2016). Cognitive impairment and rehabilitation strategies after traumatic brain injury. *Indian J. Psychol. Med.* 38 172–181. 10.4103/0253-7176.183086 27335510 PMC4904751

[B6] Benjamin E. J. Virani S. S. Callaway C. W. Chamberlain A. M. Chang A. R. Cheng S. (2018). Heart disease and stroke statistics-2018 update: a report from the American heart association. *Circulation* 137 e67–e492. 10.1161/CIR.0000000000000558 29386200

[B7] Bosboom P. R. Almeida O. P. (2016). Cognitive domains and health-related quality of life in Alzheimer’s disease. *J. Gerontol. B Psychol. Sci. Soc. Sci.* 71 275–287. 10.1093/geronb/gbu090 25098526

[B8] Bradshaw P. J. Jamrozik K. D. Gilfillan I. S. Thompson P. L. (2006). Asymptomatic long-term survivors of coronary artery bypass surgery enjoy a quality of life equal to the general population. *Am. Heart J.* 151 537–544. 10.1016/j.ahj.2005.04.007 16442928

[B9] Bruce K. M. Yelland G. W. Smith J. A. Robinson S. R. (2013). Recovery of cognitive function after coronary artery bypass graft operations. *Ann. Thorac. Surg.* 95 1306–1313. 10.1016/j.athoracsur.2012.11.021 23333061

[B10] Bruggemans E. F. (2013). Cognitive dysfunction after cardiac surgery: pathophysiological mechanisms and preventive strategies. *Neth. Heart J.* 21 70–73. 10.1007/s12471-012-0347-x 23184600 PMC3547425

[B11] Camara N. A. Obondzo K. Hantany Y. Mouni F. Z. Hicham E. O. Bouchra E. M. (2017). Quality of life in multiple sclerosis and relationship to cognitive impairment. *J. Neurol. Sci.* 381:242 10.1016/j.jns.2017.08.693

[B12] Cesar L. A. Ferreira J. F. Armaganijan D. Gowdak L. H. Mansur A. P. Bodanese L. C. (2014). Guideline for stable coronary artery disease. *Arq. Bras. Cardiol.* 103(2 Suppl. 2), 1–56. 10.5935/abc.2014s004 25410086

[B13] Chaudhury S. Saini R. Bakhla A. K. Singh J. (2016). Depression and anxiety following coronary artery bypass graft: current Indian scenario. *Cardiol. Res. Pract.* 2016:2345184. 10.1155/2016/2345184 27034884 PMC4789419

[B14] Choi J. Twamley E. W. (2013). Cognitive rehabilitation therapies for Alzheimer’s disease: a review of methods to improve treatment engagement and self-efficacy. *Neuropsychol. Rev.* 23 48–62. 10.1007/s11065-013-9227-4 23400790 PMC3596462

[B15] Chokron S. Helft G. Perez C. (2013). Effects of age and cardiovascular disease on selective attention. *Cardiovasc. Psychiatry Neurol.* 2013:185385. 10.1155/2013/185385 24455198 PMC3886577

[B16] Cropsey C. Kennedy J. Han J. Pandharipande P. (2015). Cognitive dysfunction, delirium, and stroke in cardiac surgery patients. *Semin. Cardiothorac. Vasc. Anesth.* 19 309–317. 10.1177/1089253215570062 26660055

[B17] de Tournay-Jette E. Dupuis G. Denault A. Cartier R. Bherer L. (2012). The benefits of cognitive training after a coronary artery bypass graft surgery. *J. Behav. Med.* 35 557–568. 10.1007/s10865-011-9384-y 22068879

[B18] Eriksen B. A. Eriksen C. W. (1974). Effects of noise letters upon the identification of a target letter in a nonsearch task. *Percept. Psychophys.* 16 143–149. 10.3758/bf03203267

[B19] Fabre C. Chamari K. Mucci P. Masse-Biron J. Prefaut C. (2002). Improvement of cognitive function by mental and/or individualized aerobic training in healthy elderly subjects. *Int. J. Sports Med.* 23 415–421. 10.1055/s-2002-33735 12215960

[B20] Goghari V. M. Lawlor-Savage L. (2017). Comparison of cognitive change after working memory training and logic and planning training in healthy older adults. *Front. Aging Neurosci.* 9:39. 10.3389/fnagi.2017.00039 28293187 PMC5328972

[B21] Goto T. Maekawa K. (2014). Cerebral dysfunction after coronary artery bypass surgery. *J. Anesth.* 28 242–248. 10.1007/s00540-013-1699-0 23982856

[B22] Grasso M. G. Broccoli M. Casillo P. Catani S. Pace L. Pompa A. (2017). Evaluation of the impact of cognitive training on quality of life in patients with multiple sclerosis. *Eur. Neurol.* 78 111–117. 10.1159/000478726 28738376

[B23] Gray B. E. Hahn B. Robinson B. Harvey A. Leonard C. J. Luck S. J. (2014). Relationships between divided attention and working memory impairment in people with schizophrenia. *Schizophr. Bull.* 40 1462–1471. 10.1093/schbul/sbu015 24748559 PMC4193709

[B24] Gudmundsdottir B. Beck J. G. Coffey S. F. Miller L. Palyo S. A. (2004). Quality of life and post trauma symptomatology in motor vehicle accident survivors: the mediating effects of depression and anxiety. *Depress Anxiety* 20 187–189. 10.1002/da.20037 15580574

[B25] Hammeke T. A. Hastings J. E. (1988). Neuropsychologic alterations after cardiac operation. *J. Thorac. Cardiovasc. Surg.* 96 326–331. 3260980

[B26] Harris R. Croce B. Tian D. H. (2013). Coronary artery bypass grafting. *Ann. Cardiothorac. Surg.* 2:579. 10.3978/j.issn.2225-319X.2013.07.05 23977640 PMC3741873

[B27] Hatmi Z. N. Tahvildari S. Gafarzadeh Motlag A. Sabouri Kashani A. (2007). Prevalence of coronary artery disease risk factors in Iran: a population based survey. *BMC Cardiovasc. Disord.* 7:32. 10.1186/1471-2261-7-32 17971195 PMC2200651

[B28] Hevey D. Brown A. Alison C. Helen N. Mary K. Henry H. J. (2003). Four week multidisciplinary cardiac rehabilitation product similar improvements in exercise capacity and quality of life to a 10 week program. *J. Cardiopulm. Rehabil.* 23 17–21. 10.1097/00008483-200301000-00004 12576907

[B29] Hogue C. W. Gottesman R. F. Stearns J. (2008). Mechanisms of cerebral injury from cardiac surgery. *Crit Care Clin* 24 83–98. 10.1016/j.ccc.2007.09.004 18241780 PMC2276597

[B30] Imtiaz S. A. Shah S. L. (2008). Treatment of missing values in process data analysis. *Can. J. Chem. Eng.* 86 838–858. 10.1002/cjce.20099

[B31] Järvinen O. Saarinen T. Julkunen J. Huhtala H. Tarkka Matti R. (2003). Changes in health-related quality of life and functional capacity following coronary artery bypass graft surgery. *Eur. J. Cardiothorac. Surg.* 24 750–756. 10.1016/s1010-7940(03)00413-5 14583308

[B32] Kaur M. Kumar A. Kumari V. (2013). Quality of Life and Lifestyle of Patients Before and after coronary artery bypass grafting (CABG). *IOSR J. Nurs. Health Sci.* 2 10–15.

[B33] Keizer A. M. Hijman R. Kalkman C. J. Kahn R. S. van Dijk D. Octopus Study G. (2005). The incidence of cognitive decline after (not) undergoing coronary artery bypass grafting: the impact of a controlled definition. *Acta Anaesthesiol. Scand.* 49 1232–1235. 10.1111/j.1399-6576.2005.00835.x 16146457

[B34] Kiessling A. Henriksson P. (2005). Perceived cognitive function in coronary artery disease – An unrecognised predictor of unemployment. *Qual. Life Res.* 14 1481–1488. 10.1007/s11136-005-0195-x 16110928

[B35] Knipp S. C. Matatko N. Wilhelm H. Schlamann M. Massoudy P. Forsting M. (2004). Evaluation of brain injury after coronary artery bypass grafting. A prospective study using neuropsychological assessment and diffusion-weighted magnetic resonance imaging. *Eur. J. Cardiothorac. Surg.* 25 791–800. 10.1016/j.ejcts.2004.02.012 15082284

[B36] Kok W. F. van Harten A. E. Koene B. M. Mariani M. A. Koerts J. Tucha O. (2014). A pilot study of cerebral tissue oxygenation and postoperative cognitive dysfunction among patients undergoing coronary artery bypass grafting randomised to surgery with or without cardiopulmonary bypass^*^. *Anaesthesia* 69 613–622. 10.1111/anae.12634 24750013

[B37] Lee H. T. Shin J. Lim Y. H. Kim K. S. Kim S. G. Kim J. H. (2015). Health-related quality of life in coronary heart disease in Korea: the Korea national health and nutrition examination survey 2007 to 2011. *Angiology* 66 326–332. 10.1177/0003319714533182 24792833

[B38] Leroi I. McDonald K. Pantula H. Harbishettar V. (2012). Cognitive impairment in Parkinson disease: impact on quality of life, disability, and caregiver burden. *J. Geriatr. Psychiatry Neurol.* 25 208–214. 10.1177/0891988712464823 23172765

[B39] Locke D. E. Cerhan J. H. Wu W. Malec J. F. Clark M. M. Rummans T. A. (2008). Cognitive rehabilitation and problem solving to improve quality of life of patients with primary brain tumors: a pilot study. *J. Support Oncol.* 6 383–391. 19149323

[B40] Marwick T. H. Zuchowski C. Lauer M. S. Secknus M.-A. Williams M. J. Lytle B. W. (1999). Functional status and quality of life in patients with heart failure undergoing coronary bypass surgery after assessment of myocardial viability. *J. Am. Coll. Cardiol.* 33 750–758. 10.1016/s0735-1097(98)00642-1 10080477

[B41] McKhann G. M. Goldsborough M. A. Borowicz M. Selnes O. A. Mellits E. D. Enger C. (1997). Cognitive outcome after coronary artery bypass: a one-year prospective study. *Ann. Thorac. Surg.* 63 510–515. 10.1016/s0003-4975(96)01057-0 9033329

[B42] Mirsky A. F. Anthony B. J. Duncan C. C. Ahearn M. B. Kellam S. G. (1991). Analysis of the elements of attention: a neuropsychological approach. *Neuropsychol. Rev.* 2 109–145. 10.1007/bf01109051 1844706

[B43] Mitolo M. Venneri A. Wilkinson I. D. Sharrack B. (2015). Cognitive rehabilitation in multiple sclerosis: a systematic review. *J. Neurol. Sci.* 354 1–9. 10.1016/j.jns.2015.05.004 25998261

[B44] Montazeri A. Goshtasebi A. Vahdaninia M. Gandek B. (2005). The Short form health survey (SF-36): translation and validation study of Iranian version. *Qual. Life Res.* 14 875–882. 10.1007/s11136-004-1014-5 16022079

[B45] Newman M. F. Kirchner J. L. Phillips-Bute B. Gaver V. Grocott H. Jones R. H. (2001). Longitudinal assessment of neurocognitive function after coronary-artery bypass surgery. *N. Engl. J. Med.* 344 395–402. 10.1056/NEJM200102083440601 11172175

[B46] Ojeda N. Sánchez P. Peña J. Elizagárate E. Yoller A. B. Gutiérrez-Fraile M. (2012). An explanatory model of quality of life in schizophrenia: the role of processing speed and negative symptoms. *Actas Esp. Psiquiatr.* 40 10–18. 22344491

[B47] Okrainec K. Banerjee D. K. Eisenberg M. J. (2004). Coronary artery disease in the developing world. *Am. Heart J.* 148 7–15. 10.1016/j.ahj.2003.11.027 15215786

[B48] Pan C. W. Wang X. Ma Q. Sun H. P. Xu Y. Wang P. (2015). Cognitive dysfunction and health-related quality of life among older Chinese. *Sci. Rep.* 5:17301. 10.1038/srep17301 26601612 PMC4658548

[B49] Panagopoulou E. Montgomery A. Benos A. (2006). Quality of life after coronary artery bypass grafting: evaluating the influence of preoperative physical and psychosocial functioning. *J. Psychosom. Res.* 60 639–644. 10.1016/j.jpsychores.2005.11.004 16731241

[B50] Paris A. P. Saleta H. G. de la Cruz Crespo Maraver M. Silvestre E. Freixa M. G. Torrellas C. P. (2011). Blind randomized controlled study of the efficacy of cognitive training in Parkinson’s disease. *Mov. Disord.* 26 1251–1258. 10.1002/mds.23688 21442659

[B51] Phillips-Bute B. Mathew J. P. Blumenthal J. A. Grocott H. P. Laskowitz D. T. Jones R. H. (2006). Association of neurocognitive function and quality of life 1 year after coronary artery bypass graft (CABG) surgery. *Psychosom. Med.* 68 369–375. 10.1097/01.psy.0000221272.77984.e2 16738066

[B52] Politis A. M. Norman R. S. (2016). Computer-based cognitive rehabilitation for individuals with traumatic brain injury: a systematic review. *Perspect. ASHA Special Interest Groups* 1 18–46. 10.1044/persp1.SIG2.18

[B53] Prasad S. Dhiman R. K. Duseja A. Chawla Y. K. Sharma A. Agarwal R. (2007). Lactulose improves cognitive functions and health-related quality of life in patients with cirrhosis who have minimal hepatic encephalopathy. *Hepatology* 45 549–559. 10.1002/hep.21533 17326150

[B54] Qu B. Guo H. Q. Liu J. Zhang Y. Sun G. (2009). Reliability and validity testing of the SF-36 questionnaire for the evaluation of the quality of life of Chinese urban construction workers. *J. Int. Med. Res.* 37 1184–1190. 10.1177/147323000903700425 19761703

[B55] Rantanen A. Kaunonen M. Sintonen H. Koivisto A.-M. Åstedt-Kurki P. Tarkka M.-T. (2008). Factors associated with health-related quality of life in patients and significant others one month after coronary artery bypass grafting. *J. Clin. Nurs.* 17 1742–1753. 10.1111/j.1365-2702.2007.02195.x 18592625

[B56] Rasmussen L. S. Larsen K. Houx P. Skovgaard L. T. Hanning C. D. Moller J. T. (2001). The assessment of postoperative cognitive function. *Acta Anaesthesiol. Scand.* 45 275–289. 10.1034/j.1399-6576.2001.045003275.x 11207462

[B57] Reuter E. M. Vieluf S. Koutsandreou F. Hubner L. Budde H. Godde B. (2019). A Non-linear relationship between selective attention and associated ERP markers across the lifespan. *Front. Psychol.* 10:30. 10.3389/fpsyg.2019.00030 30745886 PMC6360996

[B58] Rezapour T. DeVito E. E. Sofuoglu M. Ekhtiari H. (2016). Perspectives on neurocognitive rehabilitation as an adjunct treatment for addictive disorders: from cognitive improvement to relapse prevention. *Prog. Brain Res.* 224 345–369. 10.1016/bs.pbr.2015.07.022 26822366

[B59] Rezapour T. Hatami J. Farhoudian A. Sofuoglu M. Noroozi A. Daneshmand R. (2017). Cognitive rehabilitation for individuals with opioid use disorder: a randomized controlled trial. *Neuropsychol. Rehabil.* 29 1273–1289. 10.1080/09602011.2017.1391103 29161998

[B60] Rief W. Shedden-Mora M. C. Laferton J. A. Auer C. Petrie K. J. Salzmann S. (2017). Preoperative optimization of patient expectations improves long-term outcome in heart surgery patients: results of the randomized controlled PSY-HEART trial. *BMC Med.* 15:4. 10.1186/s12916-016-0767-3 28069021 PMC5223324

[B61] Salazar A. M. Warden D. L. Schwab K. Spector J. Braverman S. Walter J. (2000). Cognitive rehabilitation for traumatic brain injury: a randomized trial. Defense and veterans head injury program (DVHIP) study group. *JAMA* 283 3075–3081. 10.1001/jama.283.23.3075 10865301

[B62] Samuel M. Gopal R. P. (2004). recurrent angina following coronary artery surgery solutions in the current Era. *Apollo Med.* 1 12–19. 10.1016/s0976-0016(12)60034-9

[B63] Samuel R. (2008). Cognitive rehabilitation for reversible and progressive brain injury. *Indian J. Psychiatry* 50 282–284. 10.4103/0019-5545.44752 19823615 PMC2755148

[B64] Sandi C. (2013). Stress and cognition. *Wiley Interdiscip Rev. Cogn. Sci.* 4 245–261. 10.1002/wcs.1222 26304203

[B65] Saraçlı Ö Akca A. S. Atasoy N. Önder Ö Şenormancı Ö Kaygisız İ (2015). The Relationship between quality of life and cognitive functions, anxiety and depression among hospitalized elderly patients. *Clin. Psychopharmacol. Neurosci.* 13 194–200. 10.9758/cpn.2015.13.2.194 26243848 PMC4540029

[B66] Shaw P. J. Bates D. Cartlidge N. E. French J. M. Heaviside D. Julian D. G. (1987). Long-term intellectual dysfunction following coronary artery bypass graft surgery: a six month follow-up study. *QJM Int. J. Med.* 62 259–268. 10.1093/oxfordjournals.qjmed.a068097 3498965

[B67] Sheline Y. I. Barch D. M. Garcia K. Gersing K. Pieper C. Welsh-Bohmer K. (2006). Cognitive function in late life depression: relationships to depression severity, cerebrovascular risk factors and processing speed. *Biol. Psychiatry* 60 58–65. 10.1016/j.biopsych.2005.09.019 16414031

[B68] Talamo J. Frater A. Gallivan S. Young A. (1997). Use of the short form 36 (SF36) for health status measurement in rheumatoid arthritis. *Rheumatology* 36 463–469. 10.1093/rheumatology/36.4.463 9159541

[B69] Taylor R. S. Brown A. Ebrahim S. Jolliffe J. Noorani H. Rees K. (2004). Exercise-based rehabilitation for patients with coronary heart disease: systematic review and meta-analysis of randomized controlled trials. *Am. J. Med.* 116 682–692. 10.1016/j.amjmed.2004.01.009 15121495

[B70] The Whoqol G. (1998). The World Health Organization quality of life assessment (WHOQOL): development and general psychometric properties. *Soc. Sci. Med.* 46 1569–1585. 10.1016/s0277-9536(98)00009-4 9672396

[B71] Toner I. (1998). Cerebral functional changes following cardiac surgery: neuropsychological and EEG assessment. *Eur. J. Cardio Thorac. Surg.* 13 13–20. 10.1016/s1010-7940(97)00300-x 9504725

[B72] Townsend N. Wilson L. Bhatnagar P. Wickramasinghe K. Rayner M. Nichols M. (2016). Cardiovascular disease in Europe: epidemiological update 2016. *Eur. Heart J.* 37 3232–3245. 10.1093/eurheartj/ehw334 27523477 PMC13480964

[B73] Tully P. J. Baker R. A. (2012). Depression, anxiety, and cardiac morbidity outcomes after coronary artery bypass surgery: a contemporary and practical review. *J. Geriatr. Cardiol.* 9 197–208. 10.3724/SP.J.1263.2011.12221 22916068 PMC3418911

[B74] Velligan D. I. Kern R. S. Gold J. M. (2006). Cognitive rehabilitation for schizophrenia and the putative role of motivation and expectancies. *Schizophr. Bull.* 32 474–485. 10.1093/schbul/sbj071 16641424 PMC2632243

[B75] Ware J. E. Sherbourne C. D. (1992). The MOS 36-item short-form health survey (SF–36): I conceptual framework and item selection. *Med. Care* 30 473–483. 10.1097/00005650-199206000-00002 1593914

[B76] Westaby S. Saatvedt K. White S. Katsumata T. van Oeveren W. Halligan P. W. (2001). Is there a relationship between cognitive dysfunction and systemic inflammatory response after cardiopulmonary bypass? *Ann. Thorac. Surg.* 71 667–672. 10.1016/s0003-4975(00)02405-x 11235725

[B77] Wetherell J. L. Reynolds C. A. Gatz M. Pedersen N. L. (2002). Anxiety, cognitive performance, and cognitive decline in normal aging. *J. Gerontol. B Psychol. Sci. Soc. Sci.* 57 246–255. 10.1093/geronb/57.3.p246 11983736

[B78] Woods B. Aguirre E. Spector A. E. Orrell M. (2012). Cognitive stimulation to improve cognitive functioning in people with dementia^*^. *Cochrane Database Syst. Rev.* 2:CD005562. 10.1002/14651858.CD005562.pub2 22336813

[B79] Yang P. L. Huang G. S. Tsai C. S. Lou M. F. (2015). Sleep quality and emotional correlates in Taiwanese coronary artery bypass graft patients 1 week and 1 month after hospital discharge: a repeated descriptive correlational study. *PLoS One* 10:e0136431. 10.1371/journal.pone.0136431 26291524 PMC4546334

[B80] Yoo C. Yong M. H. Chung J. Yang Y. (2015). Effect of computerized cognitive rehabilitation program on cognitive function and activities of living in stroke patients. *J. Phys. Ther. Sci.* 27 2487–2489. 10.1589/jpts.27.2487 26355244 PMC4563296

[B81] Zarghi A. (2014). Functional neurosurgery and neuro-cognitive rehabilitation. *Int. Clin. Neurosci. J.* 1 43–47. 10.22037/icnj.v1i2.7243 25337334

